# Chimeric peptide supramolecular nanoparticles for plectin-1 targeted miRNA-9 delivery in pancreatic cancer

**DOI:** 10.7150/thno.38327

**Published:** 2020-01-01

**Authors:** Ying Wu, Yuexiao Tang, Shangzhi Xie, Xiaoxiao Zheng, Shufen Zhang, Jiayan Mao, Baoming Wang, Yuerou Hou, Liqiang Hu, Kequn Chai, Wei Chen

**Affiliations:** 1 Cancer Institute of Integrated Traditional Chinese and Western Medicine, Zhejiang Academy of Traditional Chinese Medicine, Tongde Hospital of Zhejiang province, Hangzhou 310012, China; 2 Department of Genetics, Institute of Genetics, Institute of Cell Biology, Zhejiang University School of Medicine, Hangzhou 310058, China

**Keywords:** PDAC, miR-9, nanoparticle, eIF5A2, autophagy

## Abstract

Pancreatic ductal adenocarcinoma (PDAC) is a highly lethal disease with poor prognosis. Insights into the roles of MicroRNAs (miRNAs) in diseases, particularly in cancer, have made miRNAs attractive tools and targets for novel therapeutic approaches.

**Methods:** Here, we employed a novel chimeric peptide supramolecular nanoparticle delivery system for plectin-1 (PL-1)-targeted PDAC-specific miR-9 delivery *in vitro* and in pancreatic cancer patient-derived xenograft (PDX) model. RT-PCR and immunohistochemistry (IHC) were conducted to detect the expression pattern of eIF5A2. mRFP-GFP-LC3 fluorescence microscopy and Western blot were carried out to determine autophagy. Luciferase reporter assays were performed to elucidate the regulatory role of miR-9/eIF5A2 axis.

**Results:** PL-1/miR-9 nanocomplexes dramatically improve the anticancer effect of doxorubicin through downregulating eIF5A2 expression to inhibit autophagy and induce apoptosis in PDAC therapy* in vivo*. Mechanistically, miR-9 directly targets the *eIF5A2* transcript by binding to its 3'-untranslated region (3'-UTR) to reduce the expression levels and the secreted protein of eIF5A2 in PDAC cells.

**Conclusion:** PL-1/miR-9 nanoparticles can be used as a novel promising anti-cancer strategy with tumor targeting and miR-9/eIF5A2 may serve as a new potential therapeutic target for future synergic therapy against human PDAC.

## Introduction

Pancreatic ductal adenocarcinoma (PDAC) ranks amongst the most lethal cancers with a 5-year survival rate of 5-6%^1^. The lethality of pancreatic cancer is mainly due to poor prognosis and tumor relapse 2. Attempts to improve the results of PDAC treatments keep focusing on a better understanding of the pathogenesis, and developing novel strategies which could heighten the efficacy of therapy.

As a class of non-coding RNA molecules, microRNAs (miRNAs) are emerging from the “desert region” of the genome as a new source of biomarkers that could characterize disease recurrence and progression 3. MiRNAs are well appreciated to play critical roles in regulating cell differentiation, proliferation and survival by binding to complementary target mRNAs to cause mRNA translational inhibition or degradation 4, 5. Accumulative studies have revealed that miRNA dysregulation is causal in many cases of cancers, within which miRNAs act as tumor suppressors or oncogenes 6-8. Insights into the roles of miRNAs in cancers make miRNAs as attractive tools and targets for novel therapeutic approaches. Based on that, miRNA mimics and molecules which target at miRNAs exhibit as promising candidates in the development of novel clinical therapy 9-12. MiR-9 is well acknowledged to exert important roles in regulating many cellular processes, including cell differentiation, proliferation, migration, and metastasis 13-16. Notably, miR-9 is identified to display aberrant expression in various types of human cancers, suggesting an unanticipated functional versatility which is implicated in carcinogenesis 13, 17, 18. However, the function of miR-9 in chemoresistance of PDAC is largely unknown.

Insights into the roles of miRNAs in cancers make miRNAs as attractive tools and targets for novel therapeutic approaches. Based on that, miRNA mimics and molecules which target at miRNAs exhibit as promising candidates in the development of novel clinical therapy 9-12. However, clinical application of miRNA-based therapy for PDAC is hampered by many challenges, including severe off-target effects, low membrane penetrability as well as poor biological compatibility 19. Comparing to conventional strategies for miRNA delivery, using non-covalent interactions between miRNA with peptides is a promising strategy to construct supramolecular assemblies for delivery and therapy 20. Due to these, the application of new PDAC-specific miRNA delivery system could overcome these defects and provides a new strategy for miRNA intervention in chemotherapy 21. As the enhanced therapeutic efficacy, minimal toxicity, targeting ability and cell permeability 22, 23, arginine-based PTP (Plectin-1-targeting peptide)-displayed chimeric peptide-condensed supramolecular nanoparticles were employed for delivering small RNAs specifically to plectin-1-targeted pancreatic cancer cells 21. These chimeric peptides are composed of arginine-rich RNA-binding and tumor cell-targeting motifs, which endow them the ability of self-assembling via absorbing small RNAs to form supramolecular nanoparticles by electrostatic associations 21.

As an essential cellular process in maintaining homeostasis through cellular self-destruction 24, 25, autophagy has been proposed as a critical contributor to pancreatic carcinogenesis due to the requirement of high levels of autophagy in pathogenesis of PDAC 26-28. With beneficial functions for tumor cells to survive through various metabolic stresses, autophagy is indeed believed to be implicated in the development of tumor chemoresistance 29, 30. It is worthy to notice that miRNAs are considered to take part in different phases of autophagy, including phagophore induction, nucleation and expansion, and autophagosome and autolysosome maturation 31. Therefore, further understanding how miRNAs regulate the autophagy during chemotherapy will provide us more insights into the underlying molecular mechanisms of chemoresistance development.

Here we reveal a novel role of miR-9 which exerts anti-tumor effects on PDAC and high levels of miR-9 exacerbate the efficacy of doxorubicin in the chemotherapy for PDAC. Increased miR-9 efficiently gave rise to the chemo sensitivity of PDAC cells to doxorubicin via inhibiting autophagy. In addition, we found that the miR-9 represses the expression of eIF5A2 by directly binding to its 3'-untranslated region (3'-UTR) to exert its inhibiting effect on autophagy. Furthermore, the utilization of PL-1/miR-9 nanocomplexes dramatically amplified the anticancer effects of doxorubicin through reducing autophagy as well as promoting apoptosis of PDAC cells in the PDX animal model. Thus, our study provides a new potential therapeutic target for clinical therapy and demonstrates a novel promising anti-cancer strategy by the using of PL-1/miR-9 nanoparticles against PDAC.

## Methods and Materials

### Peptide, miRNA and siRNA synthesis

Peptides were synthesized by solid-phase synthesis and purified by high-performance liquid chromatography (GL Biochem Ltd, Shanghai, China). The nine C-terminal arginine residues were D-arginine. HCC-specific SP-94dr peptide was used as a control. miR-9 mimic, miR-9 inhibitor and eIF5A2 siRNA were synthesized by GenePharma Co. Ltd (Shanghai, China). For some experiments, miRNA with Cy3 or Cy5 labels at the 5' end of the sense strand was used. And the above sequences were as followed:

SP-94dr: NH2-**SFSIIHTPILPL**GGGG*RRRRRRRRR*-COOH; PL1: NH2- **KTLLPTP**GGGG*RRRRRRRRR*-COOH; miR-9 NC: UUCUUCGAACGUGUCACGUTT; miR-9 mimic: UCUUUGGUUAUCUAGCUGUAUGA; miR-9 inhibitor: UCAUACAGCUAGAUAACCAAAGA; eIF5A2 siRNA: sense 5'-GCAGACGAAAUUGAUUUCATT-3' and antisense 5'-UGAAAUCAAUUUCGUCUGCTT-3'.

### Drugs, Antibodies and Reagents

Doxorubicin (D1515), chloroquine (C6628) and Rapamycin (V900930) were purchased from Sigma-Aldrich (St. Louis, MO, USA). P62 (#39749), LC3I/II (#3868) and β-Actin (#3700) primary antibodies for Western blot were obtained from Cell Signaling Technology (Danvers, MA, USA). eIF5A2 (ab126735) and Ki67 (ab15580) were purchased from Abcam (Cambridge, MA, USA). Lipofectamine 2000 (11668019) was gained from Invitrogen (Waltham, MA, USA) and used according to the manufactures' introduction.

### Clinical PDAC specimens

Sixteen patients underwent curative resection in the Second Affiliated Hospital of Zhejiang University School of Medicine with PDACs between 2016 and 2018, and samples from these patients were used for quantitative RT-PCR. This project was approved by the Ethics Committee of Second Affiliated Hospital of Zhejiang University School of Medicine. All samples were anonymously coded in accordance with local ethical guidelines (as stipulated by the Declaration of Helsinki), and written informed consent was obtained.

### Cell lines and Cell culture

Human PDAC cell lines (CFPAC-1, PANC-1 and CAPAN-1) and lung cancer cell lines (NCI-H1299 and A549) were purchased from the American Type Culture Collection (ATCC, USA). PANC-198^32^ was presented by Prof. Zhu. CFPAC-1 cells were cultured in high glucose DMEM (Gibco, Carlsbad, CA, USA) containing 10% fetal bovine serum (FBS, Gibco) and 1% penicillin/streptomycin (Sigma-Aldrich). PANC-1, CAPAN-1, PANC-198, NCI-H1299 and A549cells were cultured in RPMI 1640 Medium (Gibco) supplemented with 10% FBS and 1% penicillin/streptomycin. Cells were maintained at 37°C in a humidified incubator with 5% CO_2_.

### Gel retardation assay

miR-9 (20 μM) in RNase-free distilled water was complexed with PL-1 peptides, at different molar ratios (PL1/miR-9) from 0 to 30 and incubated for 10 min at room temperature to form the nanoparticles. Agarose gel electrophoresis assay (3% agarose with ethidium bromide) was used to evaluate the nanocomplexes formation. Electrophoresis was done at 30 V for 30 min in TAE buffer (20 mM Tris-HCl, 10 mM glacial acetic, 0.5mM EDTA, pH 8.0), and the naked miR-9 bands on the gel was visualized under a UV transilluminator at a wavelength of 365 nm using gel imaging system (Alphalmager HP, San Francisco, CA, USA).

### Nanoparticles Characterization

The hydrodynamic diameters and zeta-potentials of the PL-1/miR-9 nanoparticles in RNase-free distilled water were measured at 25 ºC using a laser particle analyzer (Fritsch ANALYSETTE 22, Leipzig, Germany). The miR-9 sample solution at a concentration with 5 μM (PL-1/miR-9 = 20:1) was deposited onto a 300-mesh copper grid coated with carbon. The surface water was removed, and the samples were allowed to air-dry. Positive staining was performed using a 4 wt % aqueous uranyl acetate solution. Images were recorded by Transmission electron microscopy (TEM, TECNAL 10, Philips, Eindhoven, Holland).

### Nanoparticles stability Assays

miR-9 (20 μM) was mixed with PL-1 at a molar ratio of 20:1 (PL-1: miR-9) and incubated at room temperature for 10 min. For RNase A degradation stability, 1 μL of RNase A (diluted to 1 ng/μL, Takara, Japan) was added and incubated at 37 ºC with time course. For serum stability assay, mouse serum was added at a final concentration 50% (v/v), the samples were incubated at 37 ºC with time course. 3 μL samples were frozen with liquid nitrogen and stocked at -80 ºC. Before loading to agarose gel electrophoresis, the samples were treated with heparin (10 μg) to release intact miR-9. Naked miR-9 was used as a control.

### Western blot Assay

Western blotting was performed as previously described 33. In brief, cell lysates were prepared by RIPA buffer (Beyotime, shanghai, China) supplemented with a PMSF inhibitor (Beyotime, shanghai, China), and quantitative analyzed with a BCA kit (Thermo scientific, MA, USA). Protein extracts were separated by SDS-polyacrylamide gel electrophoresis (SDS-PAGE) and transferred onto a polyvinylidene difluoride (PVDF) membrane filter. After incubation with the desired antibodies, the blots were developed with Thermo Scientific's SuperSignal West Pico Chemiluminescent substrate or Millipore's Immunobilon Western Chemiluminescent HRP substrate.

### Quantitative RT-PCR analysis

Total RNA was isolated from tissues or cells using a miRNeasy Mini Kit (Qiagen, Germany), according to the manufacturer's protocol. Reverse transcription of total miRNA and mRNA was performed using a miScript Reverse Transcription Kit (Qiagen, Germany). RT-PCR was performed using the miScript SYBR Green PCR Kit (Qiagen, Germany) and QuantiTect SYBR Green PCR Kit (Qiagen, Germany). β-Actin and U6 was utilized as an internal control for normalization. The oligonucleotide primers used are as follows: Human eIF5A2: forward primer 5'-TATGCAGTGCTCGGCCTTG-3' and reverse primer 5'-TTGGAACATCCATGTTGTGAGTAGA-3'; Human β-Actin: forward primer 5'-TGGCACCCAGCACAATGAA-3' and reverse primer 5'-CTAAGTCATAGTCCGCCTAGAAGCA-3'; miR-9-5p: TCTTTGGTTATCTAGCTGTATGA; U6: forward primer 5'-CTCGCTTCGGCAGCACA-3' and reverse primer 5'-AACGCTTCACGAATTTGCGT-3'.

### Cell Viability and proliferation Assays

Cell viability was assessed through a cell counting kit-8 (CCK-8; KeyGEN, Nanjing, China) assay, in accordance with the manufacturer's instructions. Cell proliferation was assayed using the Click-iT 5-ethynyl-20-deoxyuridine (EdU) Imaging Kit (Invitrogen, Carlsbad, CA, USA), following the manufacturer's instructions, and counterstained with Hoechst 33342 (Invitrogen). For each replica, at least 7 fields were quantified using intensity quantification function in Image J software. Results are from three independent experiments.

### Cellular uptake Assay

Cells were seeded on glass coverslips and incubated for overnight. Cy3 labeled miR-9/PL-1 (50 nM) were added and incubated for 6 h, cells were cultured in freshly replaced medium for further 24 h. The nuclei and endosomes/lysosomes were stained with Hoechst 33342 and FITC-labeled Dextran (Sigma-Aldrich), respectively. Cells were analyzed by CLSM or flow cytometry to determine the localization of miR-9 inside the cells.

### Histologic, TUNEL and immunohistochemical analyses

Paraffin-embedded tissue sections were subjected to H&E staining, TUNEL analysis, and immunohistochemistry (IHC). Cellular apoptosis was analyzed using the in-situ cell death detection kit, Fluorescein TUNEL System (Roche, Basel, Switzerland) according to the manufacturer's instructions. Apoptotic signals in tissue sections were visualized by fluorescence microscopy. Immunohistochemical analyses of Ki-67 and eIF5A2 were performed. Briefly, slices were permeabilized with blocking buffer (5% BSA/0.25% TX-100 in PBS) and incubated with the Ki-67 or eIF5A2 antibody overnight at 4°C. After washing with PBS, samples were incubated with HRP-conjugated secondary antibody (ZSGB-bio, Beijing, China) before analysis by microscopy. Ki67-, eIF5A2- and TUNEL-positive cells were quantified using Image J software.

### Animal experiments

Male nude mice (Shanghai Experiment Animal Centre; Shanghai, China) were raised under pathogen-free conditions with irradiated fodder. For this study, the pancreatic tumor model was established from Patient-Derived Xenografts (PDXs, The Second Affiliated Hospital of Zhejiang University, ID: 01876701), as described previously 34. Nude mice were randomly subdivided into four groups and 1 mm^3^ tumor tissues from PDX mice were injected into the right axillary fossa of each mouse. Xenograft length (L) and width (W) were measured with sliding caliper every two days, and tumor volumes were calculated using the formula (L×W^2^)/2. The body weight of each mouse was also recorded every two days. When tumor volumes reached 60-100 mm^3^, the four subgroups were subjected to different treatments: saline (control), doxorubicin (2 mg/kg body weight), doxorubicin plus SP-94dr/miR-9 nanoparticles, and doxorubicin plus PL-1/miR-9 nanoparticles (equal volume of diluents). Doxorubicin was administered intraperitoneally, and nanoparticles were injected intravenously every two days for two weeks. After two weeks of treatment, mice were euthanized by cervical dislocation, the xenografts were dissected from each mouse and tumor weights were measured. The tumor regression rate was calculated as the mean tumor weight of the experimental group/mean tumor weight of the control group×100%.

For the IVIS image assay, 12 PDXs-bearing nude mice were randomly subdivided into three groups, and were sacrificed 24 h after intravenous injection of normal saline, PL-1/miR-9 nanoparticles or SP-94dr/miR-9 nanoparticles. The xenografts were visualized, and the near infrared fluorescence (NIRF) imaging of Cy5 labeled miR-9 nanoparticles were obtained with an IVIS Spectrum Preclinical *In vivo* Imaging System (PerkinElmer, Waltham, MA, USA).

All animal experiments were performed according to protocols approved by the Institutional Animal Care and Use Committee at the Second Affiliated Hospital of Zhejiang University.

### Autophagy flux analysis

Cells were transfected with mRFP-GFP-LC3 adenovirus (Hanbio Biotech, Shanghai, China) for 24 h. Then cells were treated as indicated. Treated cells were fixed with 4% paraformaldehyde in PBS, images were obtained using a laser scanning confocal microscope. Autophagy flux was evaluated by confocal counting of the cells with GFP-LC3 (green) puncta, RFP-LC3 (red) puncta and GFP^+^/mRFP^+^-LC3 (yellow) puncta. At least 50 cells were counted per sample in triplicate experiment.

### Statistical analysis

Data are presented as the mean ± standard deviation (SD). Statistical analysis was conducted using unpaired two-tailed t-test, one-way or two-way analysis of variance (ANOVA), followed by Bonferroni's posttest with GraphPad Prism 5.0. P<0.05 was considered statistically significant.

## Results

### miR-9 gave rise to the doxorubicin sensitivity of Pancreatic ductal adenocarcinoma cells

**Figure 1:**
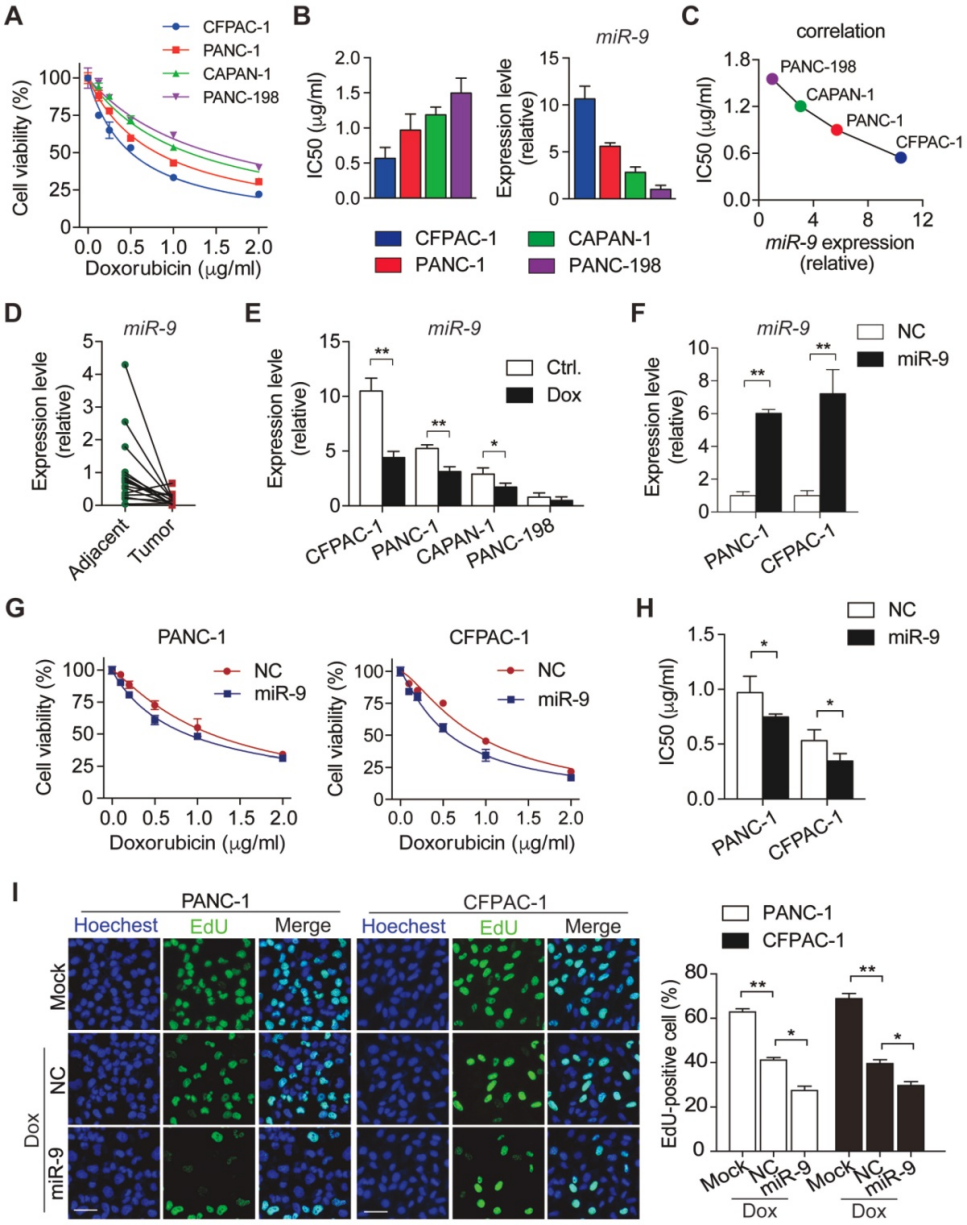
** miR-9 enhances doxorubicin sensitivity in PDAC cells. (A)** PDAC cells were incubated with indicated concentration (0, 0.125, 0.25, 0.5, 1, 2 μg/ml) of doxorubicin for 48 hr. Cell viability was assessed using Cell Counting Kit-8 assay. **(B)** Quantitative IC50 analysis of doxorubicin and quantitative RT-PCR analysis of miR-9 abundance in PDAC cells (n=3 independent experiments). **(C)** The correlation between miR-9 expression and IC50 value of PDAC cell lines for doxorubicin. **(D)** Quantitative RT-PCR analysis of miR-9 abundance in paired Adjacent and Tumor from PDAC patients (n=16). **(E)** PDAC cells were treated with 0.5 μg/ml doxorubicin for 48 hr. Shown are quantitative RT-PCR analysis of miR-9 abundance. **(F-H)** PDAC cells were treated with indicated concentration of doxorubicin for 48 hr after lipofectamine 2000 (Lipo) mediated miR-9 or control transfection in PANC-1 cells and CFPAC-1 cells. Quantitative RT-PCR analysis of miR-9 abundance (F). Cell viability was assessed using Cell Counting Kit-8 assay (G). Shown are quantitative IC50 analysis of doxorubicin (n=3 independent experiments) (H). **(I)** EdU analysis of proliferation in PDAC cells. Cells were treated with doxorubicin after lipofectamine mediated miR-9 or control transfection in PANC-1 cells and CFPAC-1 cells. Shown are representative EdU labeling images (left) and quantifications of EdU-positive cells in percentages (right), respectively. Scale bars, 50 μm. Data are presented as the mean ± SD, and analyzed with Student's *t*-test or one-way ANOVA. *P < 0.05, **P < 0.01

To characterize the sensitivity of PDAC cells to doxorubicin, panels of PDAC cells (CFPAC-1, PANC-1, CAPAN-1 and PANC-198) were treated with various concentrations of doxorubicin. After 48h incubation with doxorubicin, cell viability was determined by CCK8 assays (**Figure 1A**). Meanwhile, IC50 value of doxorubicin and miR-9 expression levels were evaluated in the four PDAC cells lines (**Figure 1B**). Interestingly, IC50 values of different cell lines for doxorubicin displayed an obviously negative correlation with miR-9 expression levels (**Figure 1C**). Furthermore, miR-9 expression was detected in 16 pairs of PDAC tumor tissues and para-tumor tissues from patients. Impressively, tumor tissues (Tumor) exhibited visually lower miR-9 expression than their paired para-tumor tissues (Adjacent) (**Figure 1D**), strongly suggesting a tumor-repressor role of miR-9 in PDAC. In addition, the obvious reductions of miR-9 expression were observed in PDAC cells after doxorubicin treatment for 48 hours (**Figure 1E**) and even longer time (**Figure S1A**). Together, these data strongly suggest that miR-9 serves as a tumor repressor and is involved in the response to doxorubicin of PDAC cells.

To investigate the physiological roles of miR-9 in the chemosensitivity of PDAC, PANC-1 and CFPAC-1 cells were transfected with miR-9 mimics to increase miR-9 levels (**Figure 1F**). Next, CCK-8 assays were performed to examine the effect of miR-9 on cell viability of PDAC cells upon doxorubicin treatments. Intriguingly, the increased miR-9 significantly reduced cell viability of both cell lines (**Figure 1G**). Additionally, heightened miR-9 levels resulted in higher chemosensitivity of PDAC cells to doxorubicin as indicated by IC50 values (**Figure 1G-H**). Consistently, with the reduction of miR-9 levels by the transfection of miR-9 inhibitor, PDAC cells exhibited heightened cell viability and dramatically enhanced chemoresistance in the context of doxorubicin treatment (**Figure S1A-C**).

To examine whether miR-9 impacts on cell growth which is inhibited upon doxorubicin, we utilized EdU incorporation assays to assess the cell proliferation of PDAC cells. Impressively, the inhibition effects of doxorubicin on the proliferation capacity were robustly amplified by the transfection of miR-9 mimics (**Figure 1I**) in both PANC-1 and CFPAC-1 cells, while no impacts were detected in the cells after transfected with miR-9 inhibitor (**Figure S1D**).

### miR-9 enhanced cancer chemosensitivity via the autophagy pathway

**Figure 2:**
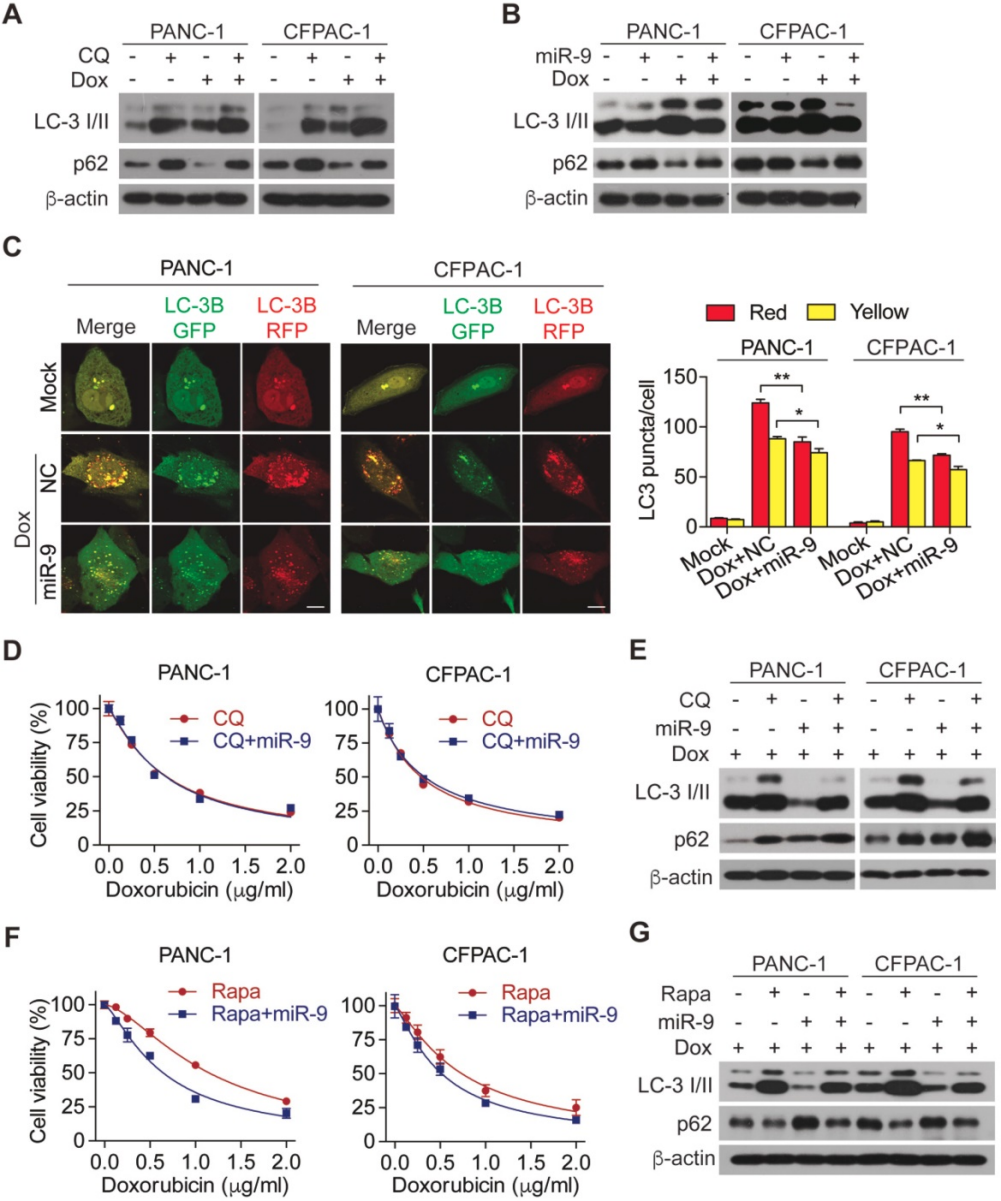
** miR-9 ameliorates doxorubicin sensitivity though inhibiting autophagy in PDAC cells. (A)** PDAC cells were incubated with 0.5 μg/ml doxorubicin for 48 hr along with or without 10 μΜ Chloroquine. LC3 and P62 protein expression were assessed using Western blot. β-actin was used as the loading control. **(B)** PDAC cells with lipofectamine mediated miR-9 or control transfection, treated with or without 0.5 μg/ml doxorubicin for 48 hr. LC3 and P62 protein expression were assessed using Western blot. β-actin was used as the loading control.** (C)** mRFP-GFP-LC3 stable PANC-1 and CFPAC-1 cells with different treatment were visualized by confocal microscopy. The numbers of GFP^+^/mRFP^+^-LC3 (yellow) and GFP^-^/mRFP^+^-LC3 (red) dots were recorded at least in 50-100 cells. Scale bars, 10 μm. **(D-G)** PDAC cells were transfected with Lipo mediated miR-9 or control, then cells were incubated with indicated concentration of doxorubicin for 48 hr along with 10 μΜ Chloroquine (D, E) or 100 nM Rapamycin (F, G). Cell viability was assessed using Cell Counting Kit-8 assay (D, F). LC3 and P62 protein expression were assessed using Western blot (E, G). β-actin was used as the loading control. Data are presented as the mean ± SD, and analyzed with Student's *t*-test, one-way or two-way ANOVA. *P < 0.05, **P < 0.01.

Linking to the promoting effects of miR-9 on chemo sensitivity of PDAC cells, we hypothesized that miR-9 is implicated in autophagy which protect cells from death under environmental pressure and is appreciated in regulating the development of doxorubicin sensitivity. To this end, chloroquine (CQ), an autophagy lysosomal inhibitor, was introduced to inhibit autophagy in PDAC cells. Impressively, the addition of CQ efficiently diminished the increased autophagy which was induced by doxorubicin (**Figure 2A**). Furthermore, blocking autophagy flux by CQ exerted an obvious promoting effect on cell viability in the presence of doxorubicin (**Figure S2A**), strongly reflecting the protective role of autophagy in the setting of chemotherapy. To explore the role of miR-9 in regulating doxorubicin-induced autophagy, the levels of LC-3 I/II as well as p62 proteins (also known as SQSTM1) were detected. Interestingly, elevated miR-9 significantly reduced the protein levels of autophagy marker LC3-II but increased p62 (**Figure 2B**). The autophagosomes were visualized by the using of mRFP-GFP-LC3, Moreover, higher miR-9 levels led to obviously reduced autophagosomes (indicated by yellow-color-labeled LC3 puncta) which was increased by the treatment of doxorubicin (**Figure 2C**). In contrast to these, decreased miR-9 by miR-9i transfection in PDAC cells caused significant autophagosome accumulation (**Figure S2B)** and upregulated LC3-II (**Figure S2C**). To further determine whether miR-9 affects doxorubicin sensitivity via regulating autophagy, we treated miR-9-overexpressed PANC-1 cells and CFPAC-1 cells with doxorubicin in combination with CQ or Rapamycin (Rapa, autophagy activator). Surprisingly, the addition of miR-9 didn't heighten the effects of CQ on either promoting chemosensitivity or autophagy in response to doxorubicin (**Figure 2D-E**). Perhaps it is the strong manual intervention of CQ on autophagy that masks the role of miR-9. In addition, the specific process of miR-9 inhibiting autophagy is different from that of CQ, which needs to be further explored. Likewise, Rapamycin significantly diminished the doxorubicin sensitivity of PDAC cells (**Figure S2D**), supporting the protective role of autophagy in chemotherapy. Notably, Rapa-induced doxorubicin resistance was significantly impaired by increased miR-9 in PDAC cells (**Figure 2F-G**). Collectively, our data demonstrates that miR-9 alleviated doxorubicin sensitivity via regulating autophagy in PDAC cells.

### miR-9 regulated autophagy via targeting *eIF5A2*

**Figure 3:**
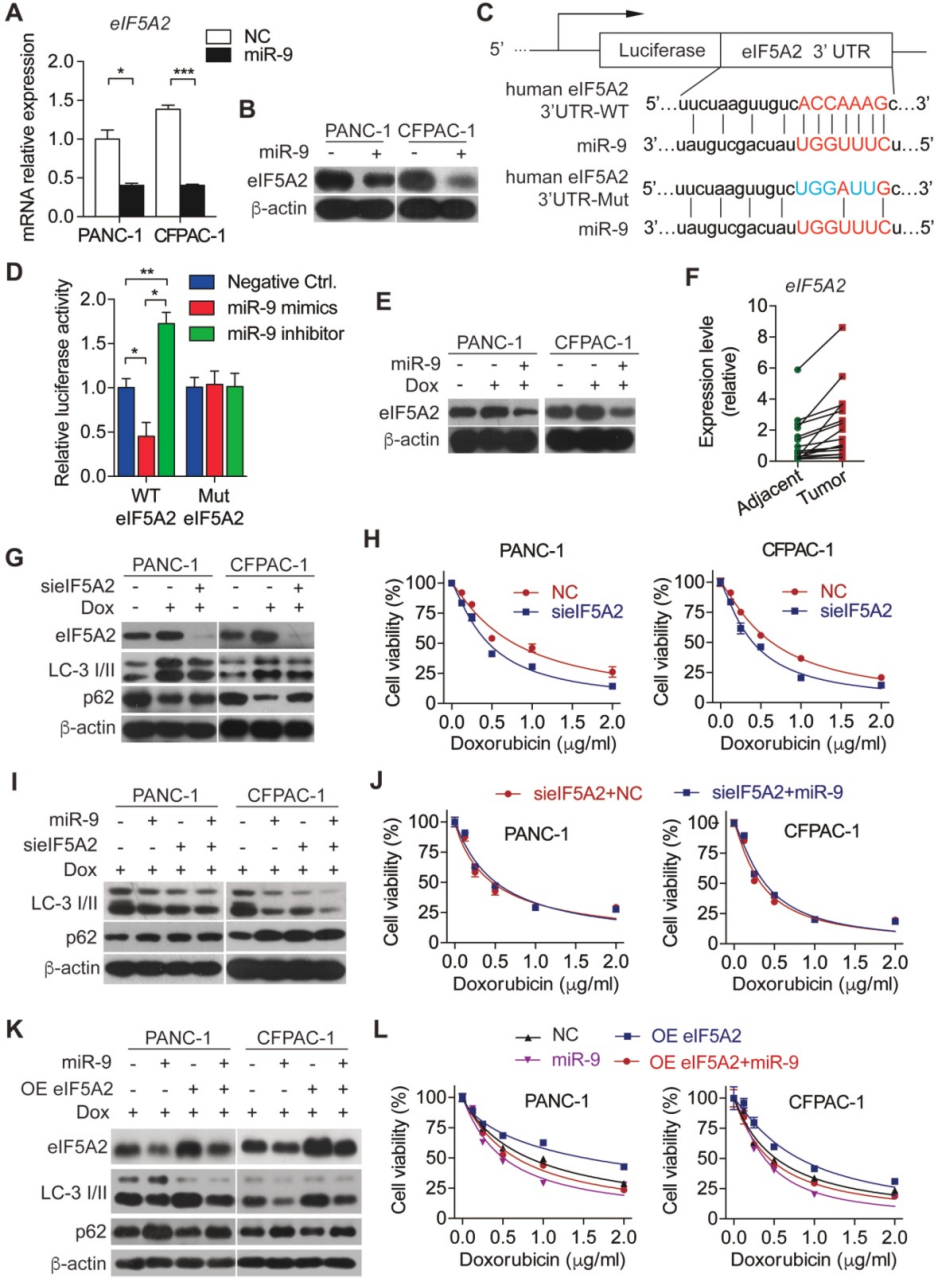
** miR-9 sensitizes PDAC cells to doxorubicin chemotherapy via directly targeting the eIF5A2 transcript. (A-B)** PDAC cells were transfected with miR-9 or control for 48 hr. Quantitative RT-PCR analysis of the mRNA abundance of eIF5A2 (A). eIF5A2 protein expression was assessed using Western blot (B). **(C)** miR-9 and its supposed binding sequence of eIF5A2, and the construct of eIF5A2-luciferase reporter plasmids (eIF5A2 WT and eIF5A2 Mut). **(D)** HEK293T cells were co-transfected for 48 h with the eIF5A2-luciferase reporter plasmids (eIF5A2 WT and eIF5A2 Mut) along with miR-9 mimics, miR-9 inhibitor or a negative control. eIF5A2 activity was determined by the luciferase assay. Shown are relative luciferase activities after normalization to Renilla that was used as the internal control. **(E)** PDAC cells with miR-9 or control transfection, treated with 0.5 μg/ml doxorubicin for 48 hr. eIF5A2 protein expression was assessed using Western blot. β-actin was used as the loading control. **(F)** Quantitative RT-PCR analysis of eIF5A2 abundance in paired Adjacent and Tumor from PDAC patients (n=16). **(G)** PDAC cells with siRNA directed against eIF5A2 or control transfection, treated with 0.5 μg/ml doxorubicin for 48 hr. eIF5A2, LC3 and P62 protein expression were assessed using Western blot. β-actin was used as the loading control. **(H)** PDAC cells with sieIF5A2 or control transfection were incubated with indicated concentration of doxorubicin for 48 hr. Cell viability was assessed using Cell Counting Kit-8 assay.** (I)** PDAC cells with miR-9 or sieIF5A2 transfection were incubated with 0.5 μg/ml doxorubicin for 48 hr. LC3 and P62 protein expression were assessed using Western blot. β-actin was used as the loading control. **(J)** PDAC cells with sieIF5A2 or miR-9 co-transfection were incubated with indicated concentration of doxorubicin for 48 hr. Cell viability was assessed using Cell Counting Kit-8 assay. **(K)** PDAC cells with miR-9 or eIF5A2 overexpression plasmid (OE eIF5A2) transfection were incubated with 0.5 μg/ml doxorubicin for 48 hr. eIF5A2, LC3 and P62 protein expression were assessed using Western blot. β-actin was used as the loading control. **(L)** PDAC cells with eIF5A2 overexpression plasmid (OE eIF5A2) or miR-9 co-transfection were incubated with indicated concentration of doxorubicin for 48 hr. Cell viability was assessed using Cell Counting Kit-8 assay. Experiments were repeated in three times. Data are presented as the mean ± SD, and analyzed with Student's *t*-test or one-way ANOVA. *P < 0.05, **P < 0.01, ***P < 0.001.

To explore the molecular mechanism underlying miR-9-regulated autophagy in PDAC cells, we searched the downstream target for miR-9 using the database of targetscan and found that eIF5A2 is a potential one. In support of this, overexpressed miR-9 led to robustly reduced eIF5A2 in both mRNA and protein levels (**Figure 3A-B**). To further elucidate the role of miR-9 in regulating *eIF5A2* expression, luciferase reporter constructs with the insertion of 3-UTR region of *eIF5A2* mRNA were applied (**Figure 3C**). Intriguingly, the overexpression of miR-9 significantly attenuated the luciferase activities, while the addition of miR-9 inhibitor gave rise to the reporter activities of eIF5A2-WT (**Figure 3D**). Blocking the interation between miR-9 and *eIF5A2* mRNA via mutating the binding site in 3-UTR of *eIF5A2* mRNA abolished the effects of miR-9 (**Figure 3D**). Additionally, the expression levels of eIF5A2 were modulated by miR-9 in both PANC-1 and CFPAC-1 cells (**Figure 3E**,**Figure S2C**). Moreover, eIF5A2 exhibited higher expression levels in PDAC tumor tissues than para-tumor tissues (**Figure 3F**), displaying an opposite pattern relative to miR-9 expression (**Figure 1D**).

Based on that, we hyposized that eIF5A2 mediates the effects of miR-9 on autophagy and doxorubicin sensitivity of PDAC cells. To this end, endogenous eIF5A2 was abolished by siRNA and attenuated doxorubicin-induced autophagy (**Figure 3G**). Consequently, the abrogation of eIF5A2 significantly gave rise to the doxorubicin sensitivity of PDAC cells (**Figure 3H**). Furthermore, the deficiency of eIF5A2 displayed no impacts on doxorubicin sensitivity improved by CQ (**Figure S3A**), but significant attenuated the doxorubicin resistance caused by Rapa (**Figure S3B**). Importantly, increased miR-9 didn't alter the impacts of eIF5A2 abrogation on autophagy and chemosensitivity of PDAC cells (**Figure 3I-J**). In contrast to these, overexpressed eIF5A2 significantly abolished the effect of miR-9 on repressing autophagy (**Figure 3K-L**), indicating that miR-9 mediates autophagy in an eIF5A2-dependent manner.

### PL1/miR-9 nanoparticles revealed a specifically targeted delivery ability and penetrability

**Figure 4:**
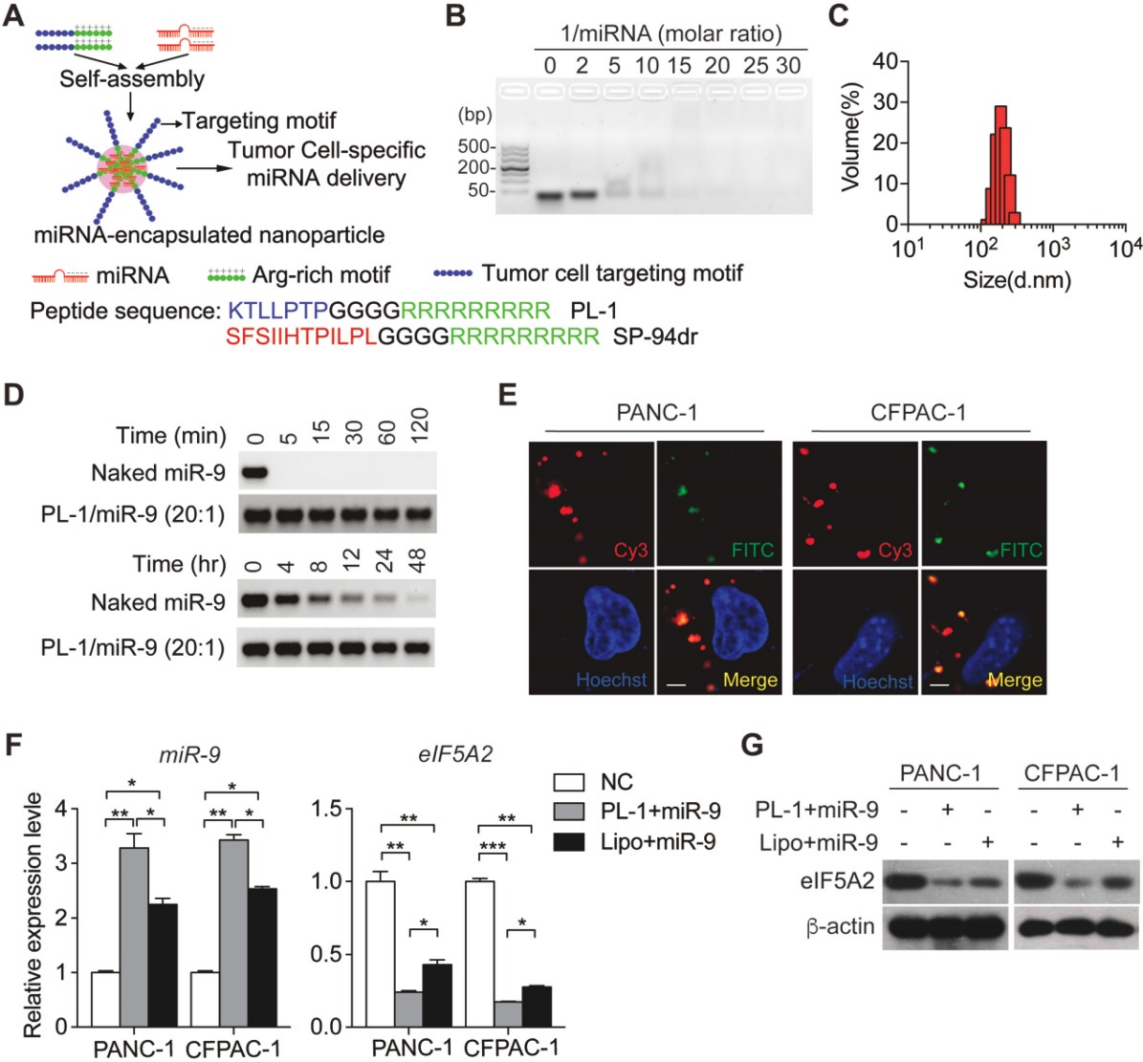
** PL-1 motif-functionalized nanoparticles are more stable and deliver miR-9 to PDAC cells with high specificity. (A)** Self-assembly of PL-1 polypeptides with miR-9 payloads to form nanocomplexes. The PDAC-specific peptide PTP was fused to the N-terminus of nine D-arginine residues via a four-glycine linker. **(B)** Agarose gel (3%) retardation assay at different molar ratios of PL-1 to miR-9.** (C)** The average hydrodynamic diameter of the nanoparticles in ddH_2_O, as assessed by DLS, was 180 ± 17 nm. **(D)** Stability of naked miR-9 and miR-9 complexed with PL-1 at a molar ratio of 20:1, after incubation with RNase A (upper). Serum stability after incubation in 50% mouse-serum solution (lower). **(E)** Representative confocal fluorescence microscopy images of PANC-1 and CFPAC-1 cells treated with Cy3 (red)-labeled miR-9 (50 nM)/PL-1 peptide complex. Nuclei and endosomes/lysosomes were stained with Hoechst (blue) and FITC-labeled Dextran (green, endo/lysosome tracker). Scale bars, 10 μm. **(F-G)** PDAC cells were transfected with miR-9 using PL-1 or Lipo for 48 hr. Quantitative RT-PCR analysis of the mRNA abundance of miR-9 and eIF5A2 (F). eIF5A2 protein expression was assessed using Western blot (G). Experiments were repeated in three times. Data are presented as the mean ± SD, and analyzed with Student's *t*-test or one-way ANOVA. *P < 0.05, **P < 0.01, ***P < 0.001.

Previously, we have established a chimeric peptide supramolecular nanoparticle for plectin-1 targeted PDAC-specific miRNA delivery system 21. This chimeric peptide composed of arginine-rich RNA-binding and PDAC targeting PTP motif (amino acid sequence: KTLLPTP), and capture polyanionic miRNA through electrostatic interactions and then form nanoparticles by self-assembly (**Figure 4A**). To test the miRNA binding ability of the designed peptides, a gel retardation assay was performed with the samples of different PL-1/miR-9 molar ratios. The retardation of naked miR-9 bands gradually increased along with PL-1/miR-9 molar ratio. When the ratio of PL-1/miR-9 was above 20:1, the naked miR-9 band was not visualized in the gel (**Figure 4B**). The hydrodynamic diameters of PL-1/miR-9 self-assembled nanoparticles at the ration of 20:1 were 180 ± 17 nm as measured by dynamic light scattering (DLS) (**Figure 4C**). These results were confirmed by direct visualization of the dried PL-1/miR-9 nanoparticles via TEM by which spherical particles of 100-200 nm in diameter were observed (**Figure S4A**). Additionally, PL-1/miR-9 nanoparticles had the zeta potential of around +12.5 mV (**Figure S4B**) and exhibited excellent solubility and stability in various solvents including ddH_2_O, PBS and cell medium (**Figure S4C**). To test whether the envelopment of nanoparticles could protect miR-9 from degradation RNase, PL-1/miR-9 nanoparticles were incubated with RNase A or in mouse serum. Compared to naked miR-9 which was completely abolished within 5 min, the levels of miR-9 inside PL-1/miR-9 nanoparticle exhibited no obvious alterations after RNase incubation as long as 120 min (**Figure 4D**, top). When incubated in mouse serum, miR-9 in the PL-1/miR-9 nanoparticle was stable even longer than 48 hr, while naked miR-9 displayed robust reduction after 4 hr incubation, reflecting the much higher stability of miRNA when packed in nanoparticles (**Figure 4D, bottom**).

To assess the targeting ability of PL-1 motif-functionalized nanoparticles on PDACs cells, various tumor cells were transfected with nanoparticles packing with Cy3-labeled miR-9 prior to flow cytometry analysis to detect Cy3-positive cells. After the nanoparticles transfection, extremely heightened proportions of Cy3-positive cells were detected in PANC-1 (86.5%) and CFPAC-1 (46.6%), compared to NCI-H1299 and A549 cells (0.1% and 0%, respectively) (**Figure S4D**). Notably, Cy3-miR-9 were observed in the cytoplasm of PANC-1 and CFPAC-1 cells, and displayed highly overlapped with endosome/lysosome tracker FITC-labeled Dextran, indicating the intake of PL-1/miR-9 nanoparticles by PDAC cells (**Figure 4E**). Together, these data demonstrate the high specificity of PL-1/miR-9 nanoparticles in targeting PDAC cells.

To evaluate the efficiency of the nanoparticles in delivery miR-9, miR-9 expression was determined in PDAC cells transfected by PL1/miR-9 nanoparticles or lipofectamine 2000-packed miR-9 (Lipo/miR-9). Impressively, the cells incubated with nanoparticles exhibited much higher miR-9 levels than that of Lipo group (**Figure 4F**). Meanwhile, the effects on repressing the expression of eIF5A2 expression also reflected that nanoparticles delivered more miR-9 into PDAC cells than Lipo (**Figure 4F-G**).

**Figure 5:**
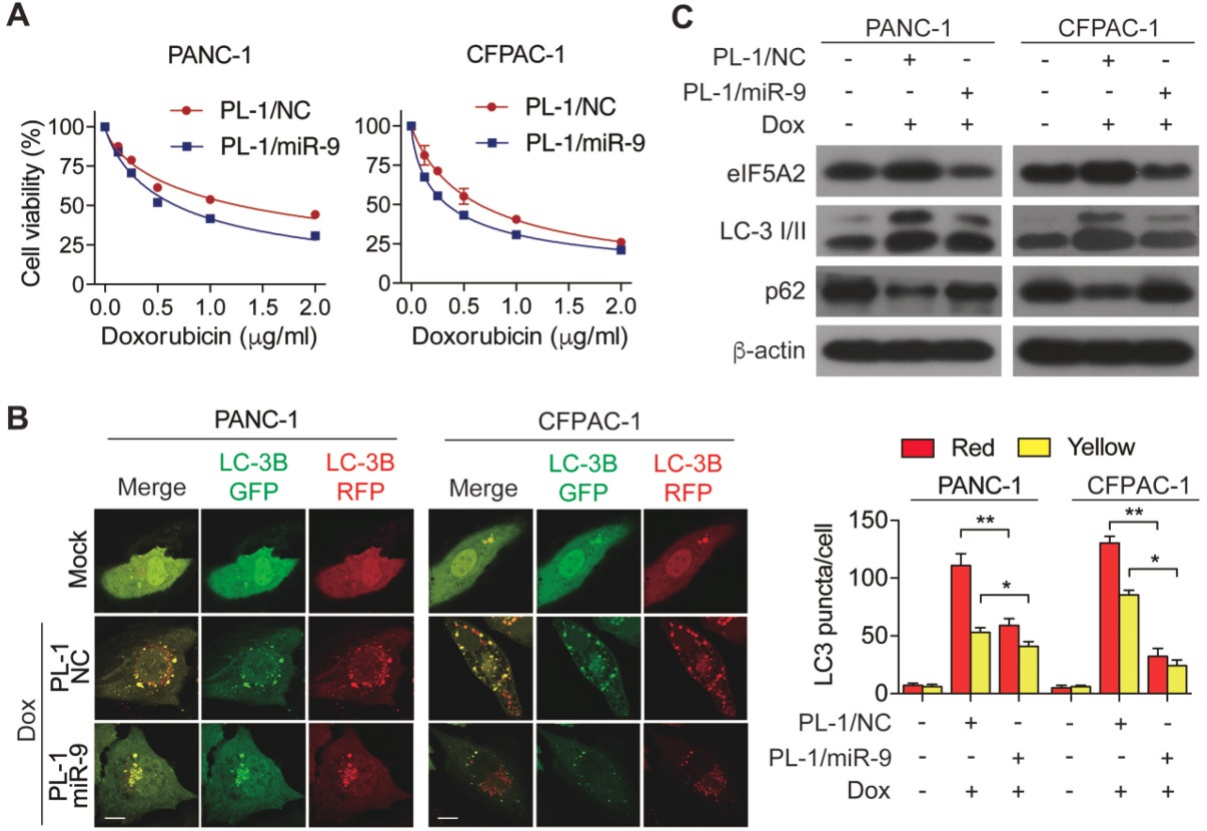
** PL-1/miR-9 nanoparticles effectively reverse the sensitivity of PDAC cells to doxorubicin. (A)** PDAC cells were incubated with indicated concentration of doxorubicin for 48 hr with PL-1/NC or PL-1/miR-9 nanoparticles pretransfection. Cell viability was assessed using Cell Counting Kit-8 assay.** (B)** mRFP-GFP-LC3 stable PANC-1 and CFPAC-1 cells with different treatment were visualized by confocal microscopy. The numbers of GFP^+^/mRFP^+^-LC3 (yellow) and GFP^-^/mRFP^+^-LC3 (red) dots were recorded at least in 50-100 cells. Scale bars, 10 μm. **(C)** PDAC cells with PL-1 mediated miR-9 or control transfection, treated with 0.5 μg/ml doxorubicin for 48 hr. eIF5A2, LC3 and P62 protein expression were assessed using Western blot. β-actin was used as the loading control. Experiments were repeated in three times. Data are presented as the mean ± SD, and analyzed with Student's *t*-test or two-way ANOVA. *P < 0.05, **P < 0.01

To determine the miR-9 delivered by nanoparticles enhanced the chemosensitivity of PDAC cells, cell viability was measured in the PL-1/miR-9 nanoparticle-transfected cells in the presence of doxorubicin. As expected, the adding of PL-1/miR-9 nanoparticles significantly gave rise to the doxorubicin sensitivity of both PANC-1 and CFPAC-1 cells (**Figure 5A**). Furthermore, relative to control groups, nanoparticle-delivered miR-9 also led to less autophagosomes formation and higher p62 protein level via reducing eIF5A2 expression (**Figure 5B**, **C**). Together, our data here indicates that PL-1/miR-9 nanoparticles successfully delivered miR-9 into PDAC cells to sensitize the cells to doxorubicin via repressing autophagy.

### PL-1/miR-9 nanoparticles enhanced the efficacy of doxorubicin therapy for PDAC *in vivo*

**Figure 6:**
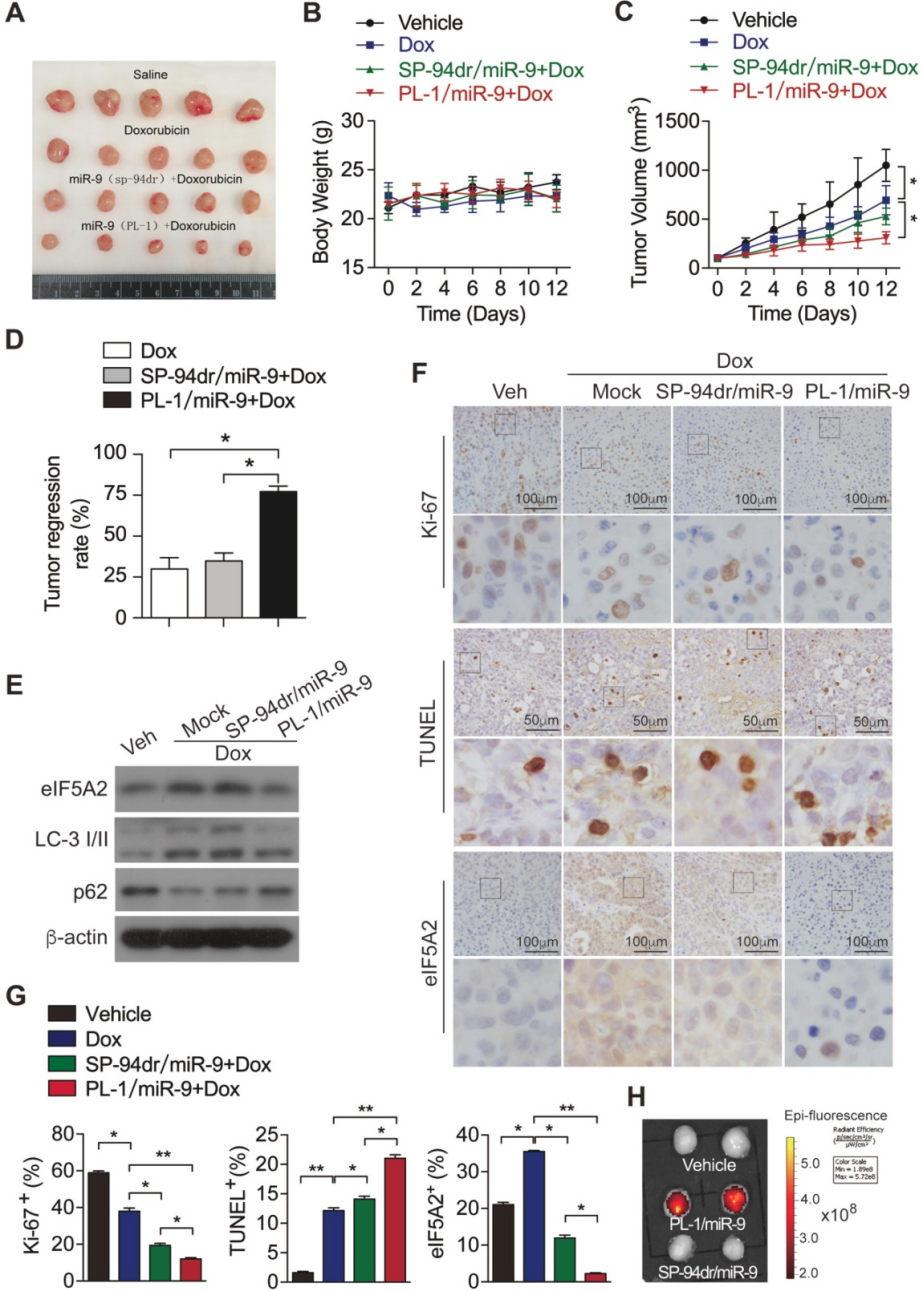
** miR-9 intervention enhances doxorubicin efficacy in xenografts growth from PDXs. (A)** Xenografts dissected from mice of different groups after two weeks of various treatments. **(B)** Body weight. **(C)** Tumor volume. **(D)** Tumor regression rates. **(E)** eIF5A2, LC3 and P62 protein expression in tumors were assessed using Western blot. β-actin was used as the loading control.** (F)** Representative TUNEL labeling images and immunohistochemistry (IHC) images of tumors stained with anti-Ki-67 or anti-eIF5A2 antibody. Scale bars, 50 μm or 100 μm. **(G)** Ki-67-positive, TUNEL-positive and eIF5A2-positive cells were quantified and are shown in percentages, respectively.** (H)** Representative *ex vivo* NIRF image of xenografts in the PDXs-bearing mice at 24 hr after intravenous injection of Vehicle (equal volume of PBS), PL-1-Cy5/miR-9 or SP-94dr-Cy5/miR-9 nanoparticles. Data are presented as the mean ± SD, and analyzed with Student's *t*-test or one-way ANOVA. *P < 0.05, **P < 0.01.

To investigate the *in vivo* effects of PL-1/miR-9 nanoparticle on doxorubicin therapy for PDAC, PDX mouse model was established as previously described 21. Next, these mice were randomly divided into 4 groups and treated respectively with: (1) vehicle control; (2) doxorubicin; (3) the combination of doxorubicin and SP-94dr/miR-9 nanoparticles; (4) the combination of doxorubicin and PL-1/miR-9 nanoparticles. Unsurprisingly, the treatment of doxorubicin for 2 weeks inhibited the growth of tumors to some extent (**Figure 6A**-**C**). SP-94 dr peptide, which is hepatocellular carcinoma cell-specific, was applied to assemble a control nanoparticle containing miR-9. Impressively, it is the miR-9-containing nanoparticles with PL-1, rather than SP-94dr, robustly augmented the anti-tumor effects of doxorubicin on the growth of PDAC xenografts (**Figure 6A**,** C**, **D**). Meanwhile, all the mice in the four groups did not exhibit any differences in body weight (**Figure 6B**). Therefore, miR-9 intervention enhanced doxorubicin efficacy in xenografts growth from PDXs. In consistent with the inhibition of miR-9 on autophagy *in vitro*, the tumors from PL-1/miR-9-treated mice displayed more p62 proteins and lower LC-3II levels (**Figure 6E**), as well as less eIF5A2 (**Figure 6E-G**) when compared to the groups of mock and SP-94dr under the doxorubicin therapy, indicating the attenuated autophagy in the xenografts after the treatment of PL-1/miR-9 nanoparticles.

To further explore the physiological functions of PL-1/miR-9 nanoparticle in repressing tumor growth, we examined the impacts on cell proliferation and apoptosis in PDAC xenografts under chemotherapy. Indeed, the doxorubicin chemotherapy combined with PL-1/miR-9 nanoparticles markedly augmented the anti-tumor effects of doxorubicin on reducing Ki-67-positive proliferating cells and increasing TUNEL-positive apoptotic cells (**Figure 6F-G**). To further confirm that the enhanced efficiency of doxorubicin therapy for PDAC is due to more miR-9 delivery by PL-1 nanoparticles, Cy5-labeled miR-9 was applied to chase miR-9 in tumors. As expected, Cy5 signals were highly enriched in the tumors of PL-1/miR-9 group relative to that of SP-94dr/miR-9 group, definitely indicating the higher affinity of PL-1 nanoparticles to PDAC than SP-94dr ones *in vivo* (**Figure 6H**).

## Discussion

Doxorubicin-based chemotherapy has been widely used to treat different types of cancers, including PDAC 35. Given the profound lethality and drug resistance to available therapies of PDAC, there is an urgent need for new approaches for clinical treatment. In this study, we identified that the levels of miR-9 were significantly downregulated in tumor tissues from PDAC patients and positively associated with doxorubicin sensitivity of PDAC cells. The increases of miR-9 in PDAC cells sensitized the tumor cells to doxorubicin via repressing autophagy. In mechanism, miR-9 targets *eIF5A2* transcript by directly binding to its 3'-UTR to exert the inhibiting effect on autophagy. Notably, we utilized PL-1/miR-9 nanocomplexes to specifically deliver miR-9 to PDAC xenograft, which dramatically amplified the anticancer effects of doxorubicin via reducing autophagy and cell proliferation, as well as promoting apoptosis of PDAC cells in the PDX animal model.

The aberrant miR-9 expression has been observed in many types of cancers, including PDAC, and exhibits quite different impact on tumor development according to the tissues or various cancer incidences by targeting different genes. Interestingly, the results of miR-9 expression are inconsistent or even contradictory in different studies 36-42. We found the obvious reductions of miR-9 expression in PDAC cells after doxorubicin treatment for 48 hours or longer. Further study presents that miR-9 can sensitize PDAC cells to doxorubicin treatment both *in vitro* and *in vivo*. In mechanism, we are the first to report that miR-9 directly targeted the mRNA of *eIF5A2* and subsequently suppressed autophagy in PDAC cells. The expression of eIF5A2 is critically required for the proliferation of some transformed cells; in particular, carcinoma cells 43. Moreover, eIF5A2 is a diagnostic marker for the presence of transformed cells in different types of tumors 44. Thus, the inhibition of eIF5A2 by miR-9 might be more specific in tumor cells than normal cells.

As one of the characteristic features of PDAC, upregulated autophagy has been acknowledged as a critical factor in promoting pancreatic carcinogenesis and chemo resistance. Linking to doxorubicin-induced autophagy exerts a protective effect on PDAC cells; inhibiting autophagy represents an intriguing target to improve outcomes for pancreatic cancer patients under chemotherapy. It is worthy to note that our findings highlight that the crucial miR-9/eIF5A2 axis enhances the anti-cancer effect of doxorubicin through regulating autophagy in PDAC cells.

Even though repressing autophagy contributes a lot to the beneficial effect of miR-9 on drug sensitivity of PDACs cells, we still cannot exclude the possibilities that other potential molecular mechanisms take part in this process. The dysregulation of ABC membrane transporters is the major obstacle to the success of cancer chemotherapies 45, 46. P-glycoprotein (P-gp), encoded by ABCB1 (also named MDR1), is highly expressed in many human tumors, where it likely contribute to resistance to chemotherapy treatment 47. It has been reported that miR-9 can bind to the 3'-UTR of ABCB1 and trigger its decay in the chemoresistant osteosarcoma cells 48. Thus, whether miR-9 improve drug resistance by regulating P-gp or other efflux pumps in the PDAC chemotherapy remain to be determined. It has yet to be further investigated whether miR-9 could affect the efflux pumps with regard to their capacity in conferring resistance to cytotoxic and targeted chemotherapy throughout the process of PDAC chemotherapy. More studies are needed to fully elucidate the molecular mechanism by which miR-9 ameliorates doxorubicin resistance.

Although a considerable number of preclinical studies involving therapeutic miRNAs have been conducted over the years, only a small part of these efforts has so far moved into clinical development. One of the biggest challenges in developing miRNA-based therapy is the identification of the best miRNA candidates or miRNA targets for each disease type. The following unovercomed challenges include the short of well-designed miRNA delivery system which could confer higher stability to the therapeutic candidate as well as more specificity to targeting cells or tissues, and meanwhile avoid cellular toxicities and off-target effects 11, 49-51. In our current study, we utilized a novel chimeric peptide supramolecular nanoparticle delivery system for PDAC-specific miR-9 delivery. Our results showed that PL-1 peptide aroused the naked miR-9 band dispersion at a molar ratio of 20:1. After mixing and self-assembly of PL-1 and miR-9, spherical particles could be clearly observed by TEM with the diameter of around 100~200 nm, which are defined as nanoparticles. It is worthy to note that the package of miR-9 in nanoparticle endows much higher stability to the miRNA even under the context of RNase A or serum incubation. Based on this, PL-1/miR-9 nanoparticles are of great benefit for miRNA devilvery as they protect the miR-9 form being degraded in circulation, which would greatly enhance its *in vivo* treatment efficacy. To elevate the specificity of nanoparticles in targeting PDAC, PTP was displayed on the surfaces of the PL-1/miR-9 nanoparticles and thus guided them to enrich in the PDAC cells. The high infinity of PTP to PDAC cells is due to its specifically binding to Plectin-1^52^, a protein which presents on the cell membrane of human and mouse PDAC cells but only stays inside normal pancreatic cells 53.

In summary, our work demonstrates that miR-9 displays positively correlated with doxorubicin sensitivity of PDAC cells and serves as a potential biomarker for predicting doxorubicin chemoresistance. Our findings also unveil that eIF5A2 dependent autophagy pathway which is mediated by miR-9 reduces doxorubicin chemosensitivity in PDAC cells. The utilization of PL-1/miR-9 nanoparticles significantly exacerbates the efficiency and specificity in miR-9 delivery *in vivo*. Our current studies provides a new and promising anti-cancer approach by using of PL-1/miR-9 nanoparticles and demonstrate miR-9/eIF5A2 as a new potential drug target for future synergic therapy against PDAC.

## Abbreviations

CCK-8: cell counting kit-8
CQ: chloroquine
DLS: dynamic light scattering
EdU: Click-iT 5-ethynyl-20-deoxyuridine
eIF5A2: Eukaryotic translation initiation factor 5A2
IC50: half maximal inhibitory concentration
IHC: immunohistochemistry
miR-9: microRNA-9
miR-9i: microRNA-9 inhibitor
PDAC: Pancreatic ductal adenocarcinoma
PDX: Patient-Derived Xenografts
PL-1: plectin-1
PTP: Plectin-1-targeting peptide
Rapa: Rapamycin
TEM: Transmission electron microscope
TUNEL: TdT-mediated dUTP Nick-End Labeling
