# Supplementary material for: Chimeric peptide supramolecular nanoparticles for plectin-1 targeted miRNA-9 delivery in pancreatic cancer
Source: Theranostics. 2020 Jan 1;10(3):1151–65. doi: 10.7150/thno.38327 (PMC6956805; doi:10.7150/thno.38327)
Supplement: Supplementary file 1 — Supplementary figures and tables. [file thnov10p1151s1.pdf]

Supplemental Information

Figure S1

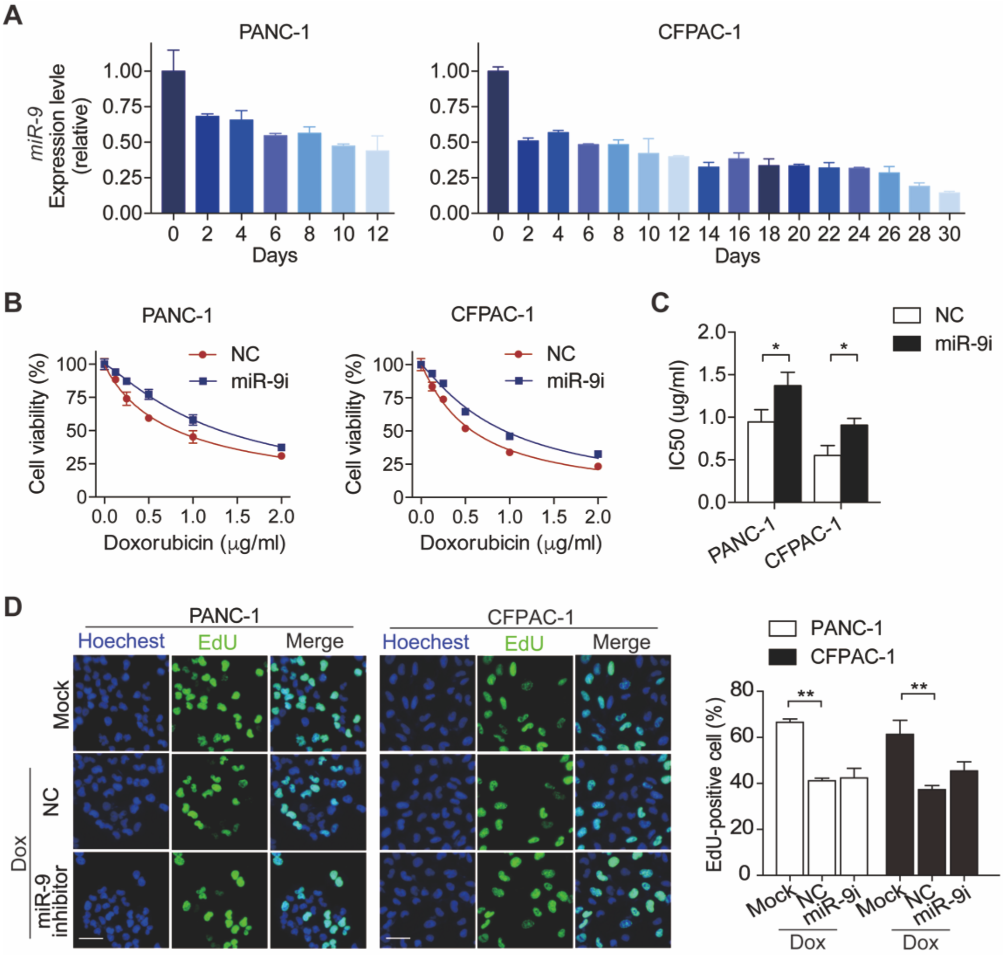

Figure S1. miR-9 inhibitor enhanced doxorubicin resistance in PDAC cells

(A) PDAC cells were incubated with 0.5 μg/ml doxorubicin for indicated time. miR-9 abundance was analyzed by quantitative RT-PCR. **(B-C)** PDAC cells were treated with indicated concentration (0, 0.125, 0.25, 0.5, 1, 2 μg/ml) of doxorubicin for 48 hr after lipofectamine mediated miR-9 inhibitor (miR-9i) or control transfection in PANC-1 cells and CFPAC-1 cells. Cell viability was assessed using Cell Counting Kit-8 assay (B). Shown are quantitative IC50 analysis of doxorubicin (n=3 independent experiments) (C). **(D)** Analysis of proliferation in PDAC cells, EdU Imaging Kit was performed. Shown are representative EdU labeling images (left) and quantifications of EdU-positive cells in percentages (right), respectively. Scale bars, 50 μm. Data are presented as the mean ± SD, and analyzed with Student's *t*-test or two-way ANOVA. *$P < 0.05$, **$P < 0.01$

Figure S2

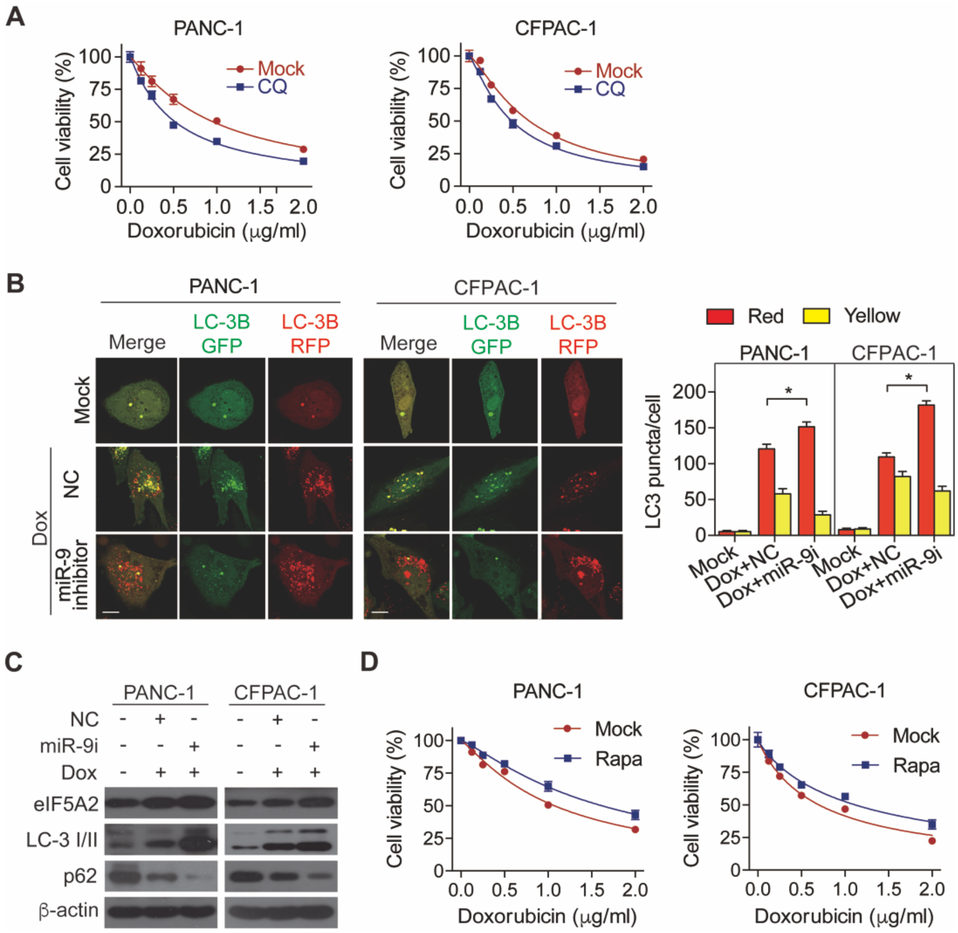

Figure S2. miR-9 inhibitor promoted autophagy in PDAC cells

(A) PDAC cells were incubated with indicated concentration of doxorubicin for 48 hr along with 10 μM Chloroquine. Cell viability was assessed using Cell Counting Kit-8 assay. **(B)** mRFP-GFP-LC3 stable PANC-1 and CFPAC-1 cells with different treatment were visualized by confocal microscopy. The numbers of $GFP^{+}/mRFP^{+}$-LC3 (yellow) and $GFP^{-}/mRFP^{+}$-LC3 (red) dots were recorded at least in 50-100 cells. Scale bars, 10 μm. **(C)** PDAC cells with lipofectamine mediated miR-9 inhibitor or control transfection, treated with 0.5 μg/ml doxorubicin for 48 hr. eIF5A2, LC3 and P62 protein expression were assessed using Western blot. β-actin was used as the loading control. **(D)** PDAC cells were incubated with indicated concentration of doxorubicin for 48 hr along with 100 nM Rapamycin. Cell viability was assessed using Cell Counting Kit-8 assay. Data are presented as the mean ± SD, and analyzed with two-way ANOVA. *$P < 0.05$

Figure S3

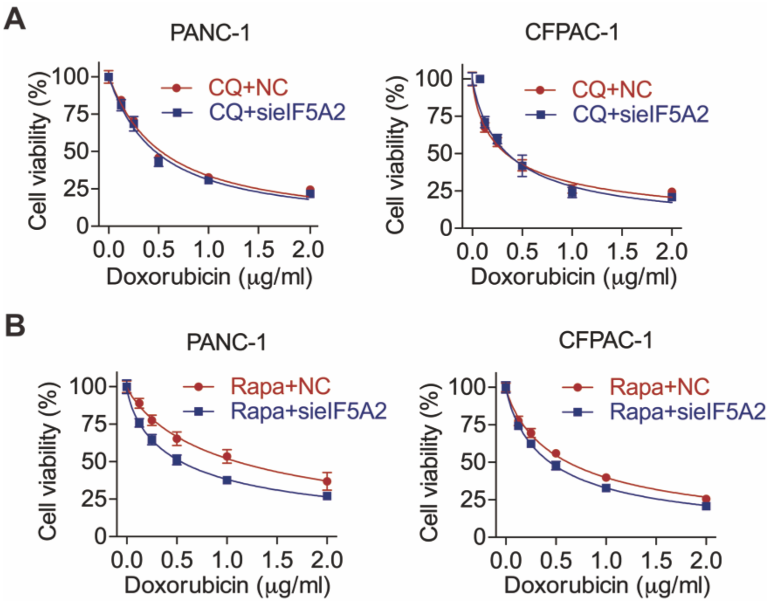

Figure S3. The impacts of eIF5A2 deficiency on doxorubicin sensitivity

(A, B) PDAC cells with sieIF5A2 or control transfection were incubated with indicated concentration of doxorubicin for 48 hr along with 10 μM Chloroquine (A) or 100 nM Rapamycin (B). Cell viability was assessed using Cell Counting Kit-8 assay. Data are presented as the mean ± SD, and analyzed with Nonlinear regression (curve fit), followed by [inhibitor] vs. normalized response equation.

Figure S4

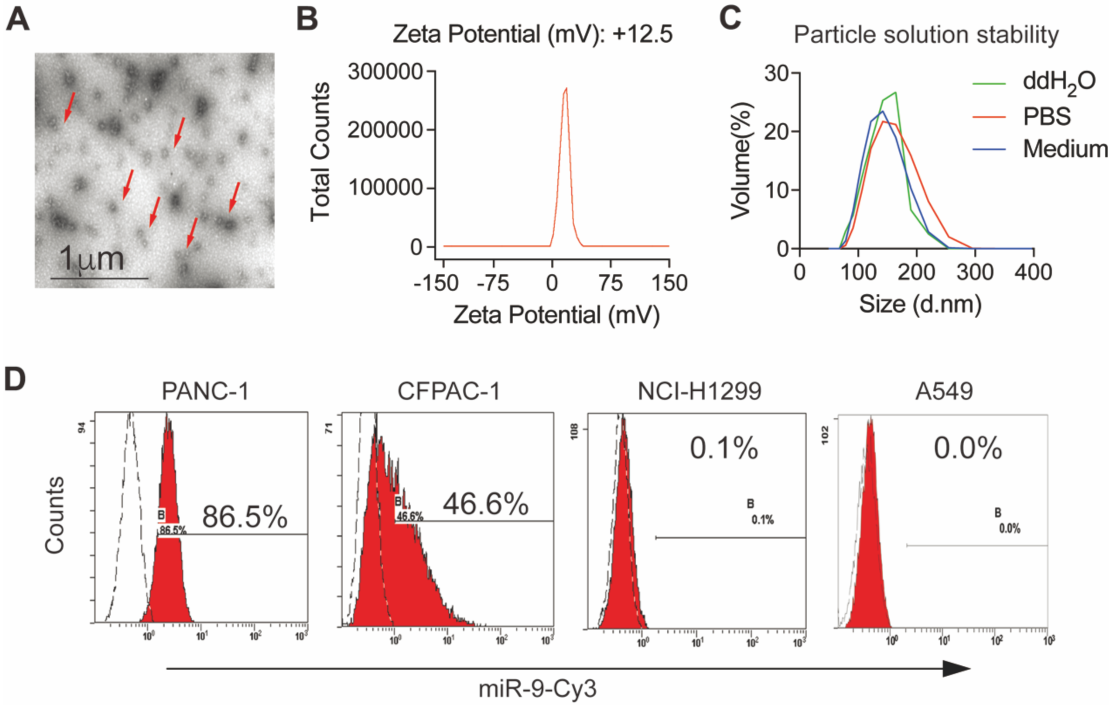

Figure S4. Characteristic property of PL-1/miR-9 nanoparticles

(A) TEM image of dried nanoparticles counterstained with 4% uranyl acetate. Scale bar, 1 μm. **(B)** zeta potential **(C)** particle solution stability **(D)** Cy3-labeled miR-9 delivery by PL-1 motif-functionalized nanoparticles into PDAC cell lines (PANC-1 and CFPAC-1) and lung cancer cell lines (NCI-H1299 and A549) assessed by flow cytometry. Experiments were repeated in three times.
